# Autophagy protects cardiomyocytes from the myocardial ischaemia-reperfusion injury through the clearance of CLP36

**DOI:** 10.1098/rsob.160177

**Published:** 2016-08-10

**Authors:** Shiguo Li, Chao Liu, Lei Gu, Lina Wang, Yongliang Shang, Qiong Liu, Junyi Wan, Jian Shi, Fang Wang, Zhiliang Xu, Guangju Ji, Wei Li

**Affiliations:** 1 Department of Radiology, State Key Laboratory of Cardiovascular Disease, Fuwai Hospital, National Center for Cardiovascular Diseases, Chinese Academy of Medical Sciences and Peking Union Medical College, Beijing 100037, People's Republic of China; 2 State Key Laboratory of Stem Cell and Reproductive Biology, Institute of Zoology, Chinese Academy of Sciences, Beijing 100101, People's Republic of China; 3 National Laboratory of Biomacromolecules, Institute of Biophysics, Chinese Academy of Sciences, Beijing 100101, People's Republic of China; 4 University of Chinese Academy of Sciences, Beijing 100049, People's Republic of China

**Keywords:** autophagy, *Atg7*, myocardial ischaemia-reperfusion injury, CLP36, stress fibre

## Abstract

Cardiovascular disease (CVD) is the leading cause of the death worldwide. An increasing number of studies have found that autophagy is involved in the progression or prevention of CVD. However, the precise mechanism of autophagy in CVD, especially the myocardial ischaemia-reperfusion injury (MI/R injury), is unclear and controversial. Here, we show that the cardiomyocyte-specific disruption of autophagy by conditional knockout of *Atg7* leads to severe contractile dysfunction, myofibrillar disarray and vacuolar cardiomyocytes. A negative cytoskeleton organization regulator, CLP36, was found to be accumulated in *Atg7*-deficient cardiomyocytes. The cardiomyocyte-specific knockout of *Atg7* aggravates the MI/R injury with cardiac hypertrophy, contractile dysfunction, myofibrillar disarray and severe cardiac fibrosis, most probably due to CLP36 accumulation in cardiomyocytes. Altogether, this work reveals autophagy may protect cardiomyocytes from the MI/R injury through the clearance of CLP36, and these findings define a novel relationship between autophagy and the regulation of stress fibre in heart.

## 1. Introduction

About one in three of all global deaths are caused by cardiovascular diseases (CVDs) each year [1,2]. Myocardial infarction (MI) serves as a major cause of cardiovascular mortality and morbidity, and the reperfusion of ischaemic hearts is a clinically effective way to cure MI [3–7]. However, when the ischaemic myocardium is reperfused with oxygen and nutrient-rich blood, some detrimental effects on clinical outcome are also accompanied, including myocardial stunning, ventricular arrhythmias and microvascular dysfunction, which are collectively referred to as myocardial ischaemia-reperfusion injury (MI/R injury) [6,8,9]. As almost half of the total myocardial damage is caused by reperfusion injury, understanding the mechanisms of this process is necessary for myocardial pathobiology and clinical treatment.

During the process of ischaemia-reperfusion, the energetic status of a cell is dynamically changed with nutrient deprivation and supply, which is related to a starvation or cellular stress-induced catabolic process, autophagy. Autophagy is a tightly regulated and conserved membrane trafficking process delivering long-lived proteins or organelles to the lysosome for degradation [10]. More than thirty autophagy-related (ATG) proteins have been characterized [11–13]. In the initiation stage of autophagosome formation, a ubiquitin-activating E1-like enzyme, ATG7, activates and promotes LC3 conjugating to the lipid/membrane by cooperating with ATG3 (E2-like enzyme) and ATG12–ATG5–ATG16 complex (E3-like enzyme). Then the LC3-lipid/membrane works as a scaffold to drive membrane expansion and double-membrane vesicle completion to form autophagosome, and the inner proteins in the autophagosome are eventually degraded once fused with lysosome [12]. Autophagy participates in many cellular processes, such as cell survival, anti-ageing, adaptation to stress conditions, intracellular quality control and biogenesis of organelles [11,14–17], and plays an important role in the pathogenesis of human diseases, in particular heart diseases [6,10,18]. For example, impaired autophagy by knocking *Atg5* out leads to cardiac hypertrophy and contractile dysfunction [19]. However, recent findings identified a paradoxical role of autophagy in MI/R injury [6,20], and the precise mechanism of autophagy regulating cardiac homeostasis remains elusive.

Stress fibres are contractile actomyosin-based bundles to provide force for a number of vital cellular processes including adhesion, migration and mechanotransduction [21,22]. Actin, myosin, actin binding proteins (ABPs) and focal-adhesion-associated proteins are the main components of stress fibres [22]. Stress fibres are commonly observed in many CVDs including cardiomyopathy, myocardial hypertrophy, as well as cardiac remodelling after MI [23,24]. Many mutations in stress fibre component proteins have been identified to be related to CVDs, such as α-actinin2 (ACTN2), myopalladin (MYPN), a-tropomyosin 1(TPM1) and so on [25,26]. Stress fibres could also incorporate α-smooth muscle actin (αSMA) in cardiac fibrosis, allowing myofibroblasts to generate increased contractile force on the matrix surrounding them [27,28]. Some stress fibre component proteins were also found in MI/R injury; however, their exact role is largely unknown.

Here, we found that cardiomyocyte-specific knockout of *Atg7* in mouse impaired autophagy process and caused severe contractile dysfunction, myofibrillar disarray and vacuolar cardiomyocytes. A negative regulator of cytoskeleton organization, CLP36, was found to be accumulated in *Atg7*-deficient cardiomyocytes. After ischaemia-reperfusion treatment, the *Atg7*-deficient mice showed aggravated MI/R injury with cardiac hypertrophy, contractile dysfunction, myofibrillar disarray and severe cardiac fibrosis, and CLP36 was found to be accumulated in ischaemia-reperfusion treated *Atg7*-deficient cardiomyocytes. Thus, our work reveals that autophagy may protect cardiomyocytes from the MI/R injury through the clearance of CLP36.

## 2. Results

### 2.1. Cardiomyocyte-specific knockout of *Atg7* in mice

**Figure 1.**
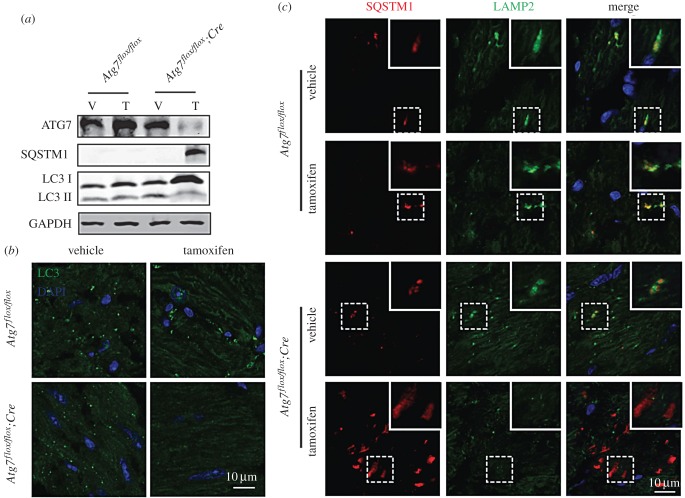
Cardiomyocyte-specific knockout of *Atg7* in mice. (*a*) The ATG7 protein level was dramatically reduced and the autophagic flux was impaired in cardiomyocyte-specific *Atg7*-deficient mouse hearts. Immunoblotting analysis of ATG7, SQSTM1 and LC3 were performed in vehicle or tamoxifen-treated *Atg7^flox/flox^* and *Atg7^flox/flox^;Cre* mouse hearts. GAPDH served as a loading control. (*b*) LC3 punctate structures disappeared in tamoxifen-treated *Atg7^flox/flox^;Cre* mouse hearts. Immunofluorescence analysis using LC3 (green) was performed in vehicle or tamoxifen-treated *Atg7^flox/flox^* and *Atg7^flox/ flox^;Cre* mouse hearts. Nuclei were stained with DAPI (blue). (*c*) SQSTM1 was accumulated and could not be sorted into the lysosome in cardiomyocyte-specific *Atg7*-deficient mouse hearts. Immunofluorescence analysis using SQSTM1 (red) and LAMP2 (green) were performed in vehicle or tamoxifen-treated *Atg7^flox/flox^*, *Atg7^flox/flox^;Cre* mouse hearts. Nuclei were stained with DAPI (blue).

To determine the functional role of autophagy in cardiomyocytes and MI/R injury, we generated temporally controlled cardiomyocyte-specific *Atg7*-deficient mice by crossing mice with a floxed *Atg7* allele to *MerCreMer* transgenic mice, which expresses the Cre recombinase in a tamoxifen-inducible and cardiomyocyte-specific manner [29,30]. These mice with both floxed *Atg7* allele and *MerCreMer* recombinase were named *Atg7^flox/flox^;Cre*. In *Atg7^flox/flox^;Cre* mice that had been treated with tamoxifen for 7 days, we observed a dramatic reduction in ATG7 protein levels in whole heart homogenates (figure 1*a*). Consistent with a role for ATG7 in autophagy [31], the protein level of membrane-associated form LC3-II was decreased and the autophagic substrate SQSTM1/p62 accumulated in *Atg7*-deficient cardiomyocytes (figure 1*a*). Immunofluorescence analysis of LC3 in mouse myocardium also showed that its punctate structures (representing autophagosomes) disappeared in tamoxifen-treated *Atg7^flox/flox^;Cre* mice (figure 1*b*). Then, we performed immunofluorescence analysis of SQSTM1 and LAMP2, a marker of lysosome [32], and found that the SQSTM1 was accumulated and could not be sorted into the lysosome in *Atg7*-deficient cardiomyocytes compared with the control groups (figure 1*c*). Although we have not got direct *in vivo* evidence, our results suggest that the autophagic flux is impaired in *Atg7*-deficient cardiomyocytes.

### 2.2. The knockout of *Atg7* in cardiomyocytes causes severe contractile dysfunction

**Figure 2.**
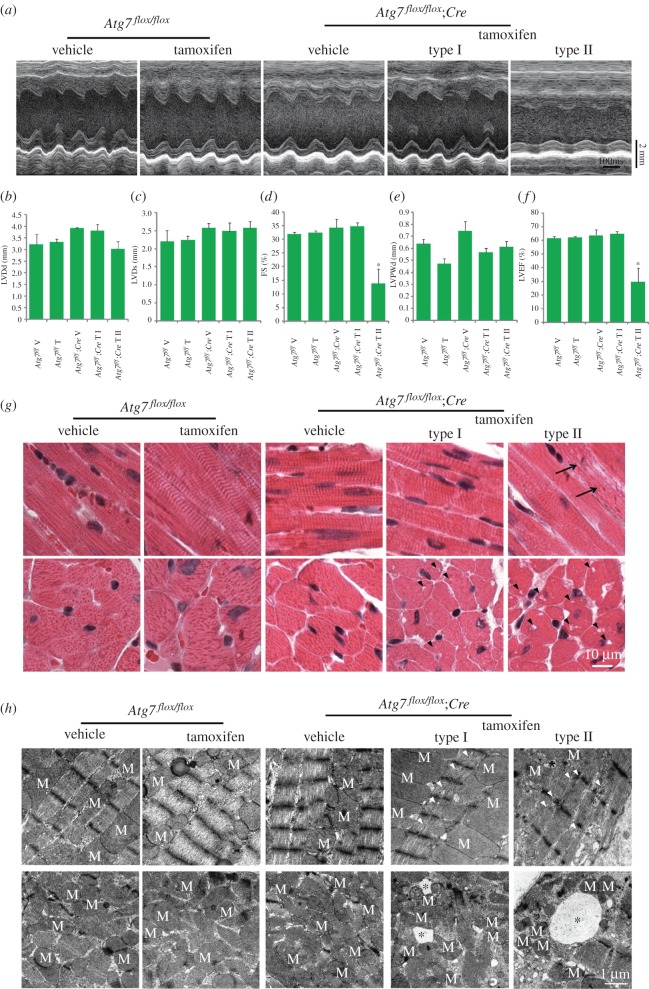
The knockout of *Atg7* in cardiomyocytes causes severe contractile dysfunction. (*a*) Representative trans-thoracic M-mode echocardiograms recorded from the parasternal short axis on the level of the papillary muscles of the left ventricle (LV) in each group. (*b–f*) The parameters of echocardiographic measurements in groups shown in (*a*). (*b*) LVDd, diastolic left ventricle internal dimension; (*c*) LVDs, systolic left ventricle internal dimension; (*d*) FS, fractional shortening of left ventricle dimension; (*e*) LVPWd, diastolic left ventricle posterior wall thickness; (*f*) LVEF, left ventricular ejection fraction. Values represent the mean ± s.e.m. of data from three to six mice in each group. *p < 0.05 versus all other groups. (*g*) The histology of the heart from vehicle or tamoxifen-treated *Atg7^flox/flox^*, *Atg7^flox/flox^;Cre* mice using hematoxylin and eosin (H&E) staining. Arrows indicate disordered myofibre, triangles indicate vacuoles in the cross-section of cardiomyocytes. (*h*) The TEM analysis of the heart from vehicle or tamoxifen-treated *Atg7^flox/flox^*, *Atg7^flox/flox^;Cre* mice. Asterisks indicate vacuoles in the cross-section of cardiomyocytes, triangles indicate disorganized myofibre, M indicates mitochondria.

To explore the role of autophagy in cardiomyocytes under baseline conditions, we first performed echocardiographic analysis of tamoxifen-treated *Atg7^flox/flox^;Cre* mice, and two types of cardiac index were identified. One type (6/9) showed normal physiological parameters, while the other (3/9) was abnormal with severe contractile dysfunction compared with control groups (figure 2*a–f*). Further, histology morphology detection of hearts from examined mice was performed by hematoxylin and eosin (H&E) staining, and *Atg7*-deficient hearts also exhibited two types, defined above. Type I showed no obvious abnormal structure in histology (i.e. no myofibrillar disarray or cardiac fibrosis) but had tiny vacuoles in the cross-section of cardiomyocytes (figure 2*g*). However, histology analysis of type II displayed that the myofibre was disorganized and larger vacuoles appeared in the cross-section of cardiomyocytes (figure 2*g*), which corresponded to echocardiographic analysis results. To further confirm it, transmission electron microscope (TEM) analysis was performed, and we found some tiny or larger vacuoles in type I and II *Atg7*-deficient hearts (figure 2*h*), respectively. The ultrastructure of myofibre was also disorganized in *Atg7*-deficient mouse hearts (figure 2*h*). Thus, we conclude that ATG7 plays important roles in the normal contraction of cardiomyocytes.

### 2.3. CLP36 is accumulated in *Atg7*-deficient cardiomyocytes

**Figure 3.**
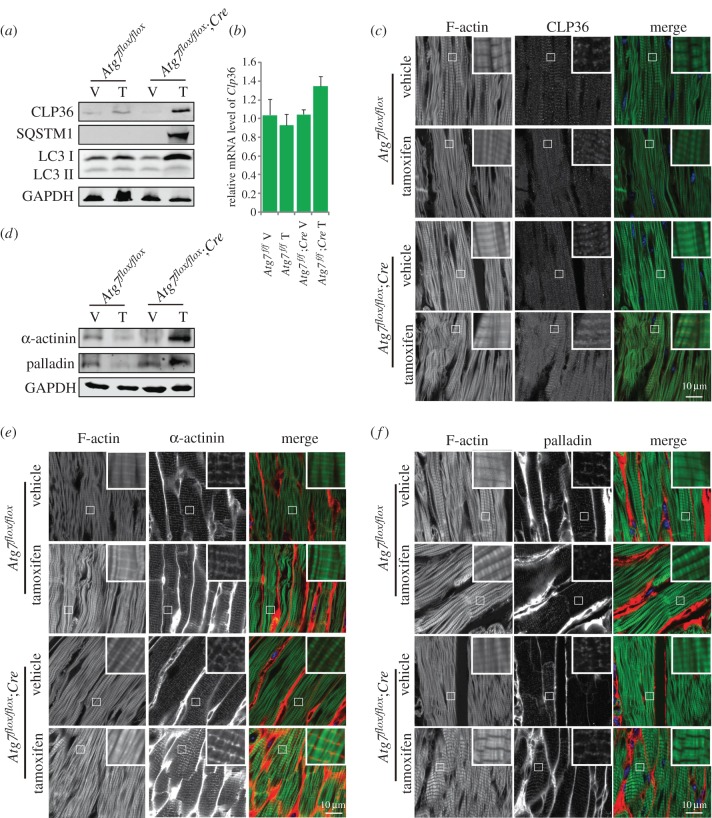
CLP36 is accumulated in *Atg7*-deficient cardiomyocytes. (*a*) The protein level of CLP36 was accumulated in *Atg7*-deficient cardiomyocytes. Immunoblotting analysis of CLP36, SQSTM1 and LC3 were performed in vehicle or tamoxifen-treated *Atg7^flox/flox^*, *Atg7^flox/flox^;Cre* mouse hearts. GAPDH served as a loading control. (*b*) Relative mRNA level of *Clp36* in vehicle or tamoxifen-treated *Atg7^flox/flox^*, *Atg7^flox/flox^;Cre* mouse hearts. (*c*) CLP36 was accumulated and localized on the Z-disc of the sarcomere in *Atg7*-deficient cardiomyocytes. Immunofluorescence analysis using phalloidin (green, labelled by FITC) and CLP36 (red) were performed in vehicle or tamoxifen-treated *Atg7^flox/flox^*, *Atg7^flox/flox^;Cre* mouse hearts. Nuclei were stained with DAPI (blue). (*d–f*) The protein level of α-actinin and palladin was accumulated in *Atg7*-deficient cardiomyocytes. (*d*) Immunoblotting analysis of α-actinin and palladin were performed in vehicle or tamoxifen-treated *Atg7^flox/flox^*, *Atg7^flox/flox^;Cre* mouse hearts. GAPDH served as a loading control. (*e*) Immunofluorescence analysis using phalloidin (green, labelled by FITC) and α-actinin (red) were performed in vehicle or tamoxifen-treated *Atg7^flox/flox^*, *Atg7^flox/flox^;Cre* mouse hearts. (*f*) Immunofluorescence analysis using phalloidin (green, labelled by FITC) and palladin (red) was performed in vehicle or tamoxifen-treated *Atg7^flox/flox^*, *Atg7^flox/flox^;Cre* mouse hearts. Nuclei were stained with DAPI (blue).

As the cytoskeleton and cytoskeleton-related proteins in cardiomyocytes are essential to ensure proper cardiac function, especially motility [33], we wondered whether the disruption of autophagy-induced contractile dysfunction was related to cytoskeleton regulation. Many studies regarding the relationship between the cytoskeleton and autophagy focused on autophagosome formation and transportation [34,35], but few studies have investigated how autophagy modulates cytoskeletal organization. Our recent work found that autophagy could promote the degradation of a negative cytoskeleton organization regulator, CLP36, to regulate cytoskeletal organization during spermatogenesis [36]. So we detected whether CLP36 might also be accumulated in *Atg7*-deficient cardiomyocytes. First, we examined the CLP36 protein level in tamoxifen-treated *Atg7^flox/flox^;Cre* mouse hearts by immunoblotting, and found that CLP36 protein was dramatically accumulated in *Atg7*-deficient mouse whole heart homogenates (figure 3*a*). To further confirm the accumulation of CLP36, which resulted from abnormal protein degradation but not the upregulated expression, we detected the relative mRNA level of *Clp36* in tamoxifen-treated *Atg7^flox/flox^;Cre* mouse hearts, and found there was no significant difference in mRNA level between tamoxifen-treated *Atg7^flox/flox^;Cre* and control groups (figure 3*b*), suggesting that the elevated CLP36 might be a result of the failure of its degradation. Then, we performed immunofluorescence analysis of CLP36 in *Atg7*-deficient heart, and found that CLP36 was localized on the Z-disc of the sarcomere and accumulated in *Atg7*-deficient mice (figure 3*b*; electronic supplementary material, figure S1). To further confirm the localization of CLP36, immunofluorescence analysis of CLP36 and Z-disc localized protein α-actinin was performed, and we found CLP36 indeed co-localized with α-actinin (electronic supplementary material, figure S2). As CLP36 could function as a cytoskeletal organization scaffold and an adaptor for the recruitment of α-actinin and palladin to form stress fibres [37], and as stress fibres are related to cardiac dysfunction [23,24], we therefore speculated whether the accumulated CLP36 enhanced the stress fibre formation and influenced cardiomyocyte contractile function in *Atg7*-deficient cardiomyocytes. To test this hypothesis, we examined the α-actinin and palladin protein level by immunoblotting and immunofluorescence in *Atg7*-deficient cardiomyocytes. Both immunoblotting and immunofluorescence results showed that α-actinin and palladin were also accumulated in *Atg7*-deficient cardiomyocytes compared with control groups (figure 3*d–f*), indicating the stress fibre formation actually increased in *Atg7*-deficient mice. Together, all these results indicate that ATG7 may participate in cardiomyocyte contractile function maintenance through the clearance of CLP36.

### 2.4. The cardiomyocyte-specific disruption of ATG7 causes CLP36 accumulation after myocardial ischaemia-reperfusion treatment

**Figure 4.**
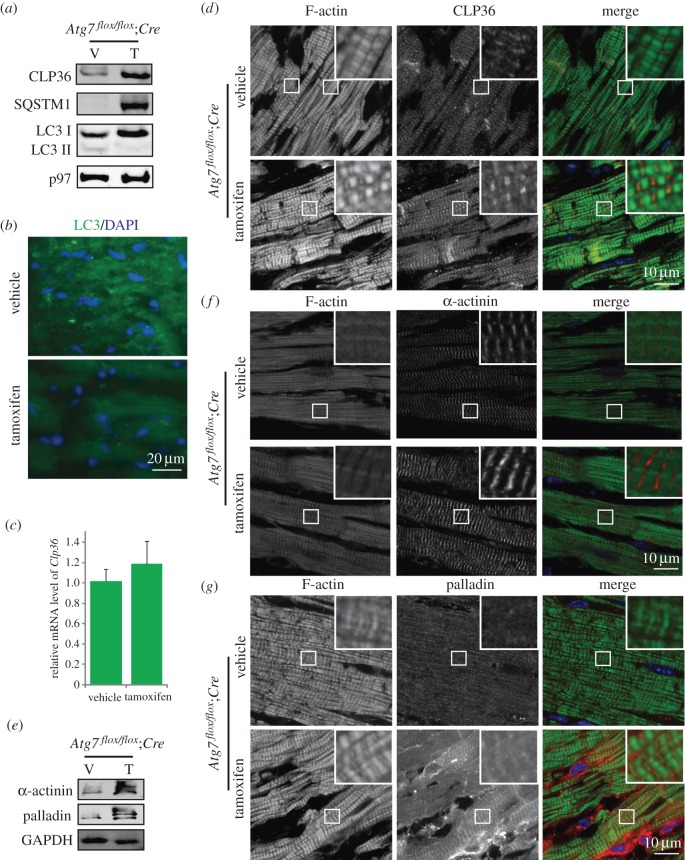
The cardiomyocyte-specific knockout of *Atg7* could also impair the autophagic flux and cause CLP36 accumulation after myocardial ischaemia-reperfusion treatment. (*a*) The CLP36 was accumulated and the autophagic flux was disrupted in ischaemia-reperfusion-treated *Atg7*-deficient mice. After myocardial ischaemia-reperfusion treatment, immunoblotting analysis of CLP36, SQSTM1 and LC3 was performed in vehicle or tamoxifen-treated *Atg7^flox/flox^;Cre* mouse hearts. p97 served as a loading control. (*b*) LC3 punctate structures disappeared in ischaemia-reperfusion-treated *Atg7*-deficient cardiomyocytes. Immunofluorescence analysis using LC3 (green) was performed in vehicle or tamoxifen-treated *Atg7^flox/flox^;Cre* mouse hearts. Nuclei were stained with DAPI (blue). (*c*) Relative mRNA level of *Clp36* in vehicle or tamoxifen-treated *Atg7^flox/flox^;Cre* mouse hearts. (*d*) Immunofluorescence analysis using phalloidin (green, labelled by FITC) and CLP36 (red) were performed in vehicle or tamoxifen-treated *Atg7^flox/flox^;Cre* mouse hearts after ischaemia-reperfusion treatment. Nuclei were stained with DAPI (blue). (*e–g*) The protein level of α-actinin and palladin was accumulated in *Atg7*-deficient cardiomyocytes after ischaemia-reperfusion treatment. (*e*) Immunoblotting analysis of α-actinin and palladin were performed in vehicle or tamoxifen-treated *Atg7^flox/flox^;Cre* mouse hearts after ischaemia-reperfusion treatment. GAPDH served as a loading control. (*f*) Immunofluorescence analysis using phalloidin (green, labelled by FITC) and α-actinin (red) were performed in vehicle or tamoxifen-treated *Atg7^flox/flox^;Cre* mouse hearts after ischaemia-reperfusion treatment. (*g*) Immunofluorescence analysis using phalloidin (green, labelled by FITC) and palladin (red) was performed in vehicle or tamoxifen-treated *Atg7^flox/flox^;Cre* mouse hearts after ischaemia-reperfusion treatment. Nuclei were stained with DAPI (blue).

To further examine the functional role of autophagy in MI/R injury under equal initial states, type I *Atg7*-deficient mice were selected to perform MI/R experiments, in which mouse hearts were exposed to ischaemia (60 min) followed by reperfusion. First, we detected the autophagic flux in tamoxifen-treated *Atg7^flox/flox^;Cre* mice after ischaemia-reperfusion treatment, and found the LC3-II reduced and the SQSTM1 accumulated (figure 4*a*) and LC3 punctate structures were disappeared in *Atg7^flox/flox^;Cre* mice (figure 4*b*), suggesting that autophagic flux was impaired. Then, we examined the protein level of CLP36 by immunoblotting and immunofluorescence analysis, and found it was also accumulated in ischaemia-reperfusion treated *Atg7*-deficient mouse hearts, but no difference in its mRNA level was found (figure 4*a,c,d*). Thus, after MI/R treatment, the cardiomyocyte-specific knockout of *Atg7* could also impair the autophagic flux and cause CLP36 accumulation. We then detected the stress fibre components in ischaemia-reperfusion-treated *Atg7*-deficient mouse hearts, and found that the α-actinin and palladin were accumulated in MI/R-treated *Atg7*-deficient mouse heart compared with their control groups (figure 4*e–g*). Therefore, we concluded that cardiomyocyte-specific disruption of ATG7 causes CLP36 accumulation and enhances stress fibre formation after MI/R treatment.

### 2.5. The cardiomyocyte-specific disruption of ATG7 aggravates the myocardial ischaemia-reperfusion injury

**Figure 5.**
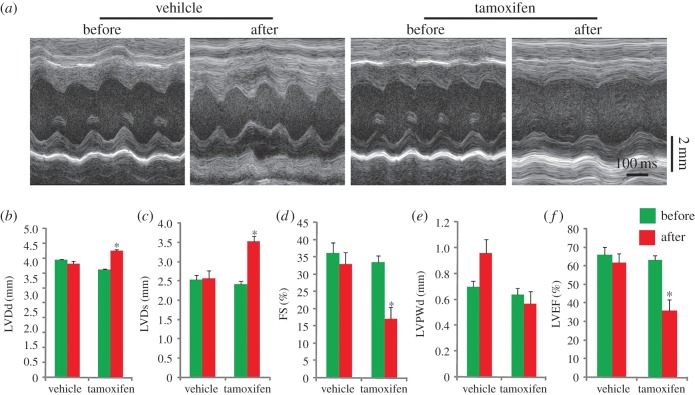
The cardiomyocyte-specific disruption of *Atg7* aggravates the myocardial ischaemia-reperfusion injury with cardiac hypertrophy and contractile dysfunction. (*a*) Representative trans-thoracic M-mode echocardiograms recorded from the parasternal short axis on the level of the papillary muscles of the left ventricle (LV) in each group before or after ischaemia-reperfusion treatment. (*b–f*) The parameters of echocardiographic measurements in (*a*). (*b*) LVDd, diastolic left ventricle internal dimension; (c) LVDs, systolic left ventricle internal dimension; (*d*) FS, fractional shortening of left ventricle dimension; (*e*) LVPWd, diastolic left ventricle posterior wall thickness; (*f*) LVEF, left ventricular ejection fraction. *p < 0.05 versus all other groups.

For further exploration of the influence of autophagy disruption after MI/R treatment, we performed echocardiographic analysis of ischaemia-reperfusion-treated *Atg7-*deficient mice. We found that the diastolic left ventricle posterior wall thickness of the *Atg7*-deficient mice was significantly increased and their ejection fraction and fractional shortening of left ventricle dimension were significantly decreased after ischaemia-reperfusion treatment (figure 5*a–f*), which indicated left ventricular dilatation and severe contractile dysfunction in ischaemia-reperfusion treated *Atg7-*deficient mice.

**Figure 6.**
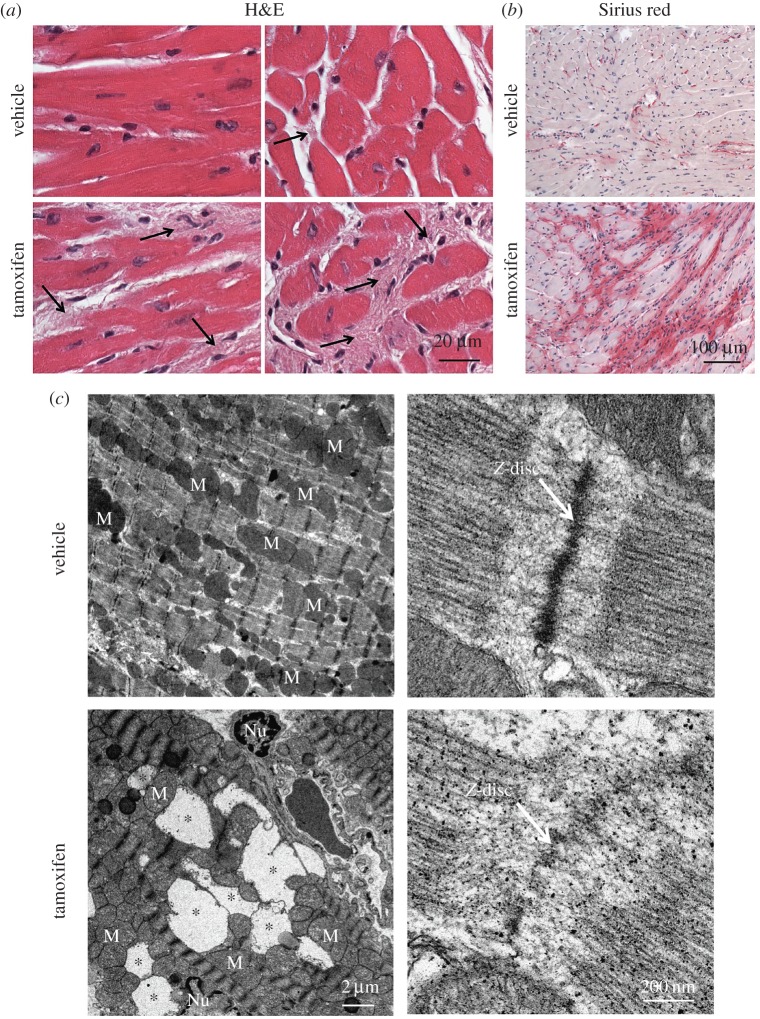
The cardiomyocyte-specific disruption of *Atg7* aggravates the myocardial ischaemia-reperfusion injury with myofibrillar disarray and severe cardiac fibrosis. (*a*) The histology of the heart from vehicle or tamoxifen-treated *Atg7^flox/flox^;Cre* mice after ischaemia-reperfusion treatment using hematoxylin and eosin (H&E) staining. Arrows indicate interstitial fibrosis. (*b*) Cardiac fibrosis detected by using Sirius-red staining in vehicle or tamoxifen-treated *Atg7^flox/flox^;Cre* mice after ischaemia-reperfusion treatment. (*c*) The TEM analysis of the heart from vehicle or tamoxifen-treated *Atg7^flox/flox^;Cre* mice after ischaemia-reperfusion treatment. Asterisks indicate vacuoles in cardiomyocytes, arrows indicate Z-disc, M indicates mitochondria, Nu indicates nucleus.

Next, we detected the histology morphology of hearts from examined mice by H&E staining, and found that the myofibres were disorganized and severe cardiac fibrosis appeared in ischaemia-reperfusion-treated *Atg7-*deficient mice (figure 6*a*). To further confirm the severe cardiac fibrosis in ischaemia-reperfusion-treated *Atg7-*deficient mice, we performed Sirius red staining, which was used to observe fibrosis levels based on the tight-binding of the stain sulfonic acid groups with the basic groups of collagen fibres [38]. We found that the area of cardiac fibrosis in ischaemia-reperfusion-treated *Atg7-*deficient mice was larger compared with that of the control group (figure 6*b*). Cardiomyocyte death is an initial event responsible for activation of fibrogenic signals in the myocardium [39], and the TEM analysis of ischaemia-reperfusion-treated *Atg7-*deficient mice showed some large vacuoles in the myocardium (figure 6*c*), suggesting that the cardiac fibrosis might be associated with cardiomyocyte loss in *Atg7-*deficient mice. In addition, we found that the sarcomere of ischaemia-reperfusion-treated *Atg7-*deficient mice was disorganized (figure 6*c*). Thus, cardiomyocyte-specific disruption of ATG7 aggravated the MI/R injury with cardiac hypertrophy, contractile dysfunction, myofibrillar disarray and severe cardiac fibrosis. As CLP36 was accumulated in *Atg7-*deficient mice and could recruit α-actinin and palladin to form stress fibres, and cardiac fibrosis was characterized by the appearance of stress fibres [37,39], so the accumulation of CLP36 might result in severe cardiac fibrosis, myofibrillar disarray and contractile dysfunction. All these results indicate that ATG7 might protect cardiomyocytes from the ischaemia-reperfusion injury through the clearance of CLP36.

## 3. Discussion

Autophagy is a catabolic recycling pathway triggered by various intra- or extracellular stimuli to maintain cellular homeostasis [40]; it has been widely characterized in cardiomyocytes, cardiac fibroblasts, endothelial cells and vascular smooth muscle cells of the cardiovascular system [41]. During cardiac development, autophagy plays an essential role in cardiac morphogenesis [42]. Under baseline conditions, autophagy has a housekeeping role in maintaining cardiac structure and cellular homeostasis in the heart [43]. Conditional knockout of *Atg5* in the heart causes a disruption in autophagy, and results in cardiac hypertrophy and contractile dysfunction [19]. The deficiency of lysosomal-associated membrane protein-2 (LAMP-2), which causes a disruption in the autophagosome–lysosome machinery, also leads to vacuolar myopathy, cardiac hypertrophy and severe cardiac dysfunction, which is known as Danon's disease [44,45]. We specifically disrupted ATG7 in cardiomyocytes, and found two types of cardiac function index and histological morphology. Type I showed normal cardiac physiological parameters, but had tiny vacuoles in the cross-section of cardiomyocytes; Type II exhibited severe contractile dysfunction and myofibrillar disarray, and mass vacuoles appeared in the cross-section of cardiomyocytes, which is in agreement with *Atg5* and *LAMP-2*-deficient mice [19,44,45]. As the phenotype of type II is more severe than type I, it was similar to the different pathogenic stages of cardiomyopathy. The difference between these two types may be caused by the knockout efficiency due to mosaic-expressed Cre recombinase. On the other hand, it has been reported that *LAMP-2*-deficient mice showed more severe symptoms than *Atg5* and *Atg7*-deficient mice [19,44,45]. As ATG5 and ATG7 participate in the canonical autophagy pathway and LAMP2 is important to the autophagosome–lysosome machinery [31,44,46], the non-canonical autophagy pathway may also be involved in maintaining cardiac structure and cellular homeostasis in the heart, and this point still requires further studies in the future.

As autophagy is upregulated in response to stresses such as nutrient deprivation, hypoxia and infection, many researches focus on the role of autophagy during cardiovascular stress, including starvation, chronic ischaemia, infarction–reperfusion injury, pressure overload, cardiomyopathy and heart failure [43]. However, autophagy functioning as a pro-survival or pro-death programme during heart disease is still controversial [43,47], especially in ischaemia-reperfusion injury [48]. Beneficial functions of autophagy during I/R could be attributed to ATP generation, protein quality control and the clearance of damaged organelles, while the detrimental effect of autophagy is excessive induction of autophagy-induced cardiomyocyte death [47,48]. However, Ma *et al*. found autophagosome clearance was markedly impaired with reperfusion, which is detrimental to cardiomyocyte survival during reperfusion. It indicates ‘impaired’ but not ‘excessive’ autophagy leads to cardiomyocyte death during ischaemia-reperfusion injury [49,50]. Recently, hydrogen sulfide, valsartan, small molecule HDAC inhibitor and intermittent fasting were also found to protect against MI/R injury by activating autophagic flux [51–54]. Here, we find that the knockout of *Atg7* in cardiomyocytes aggravates the injury with cardiac hypertrophy, contractile dysfunction, myofibrillar disarray and severe cardiac fibrosis, which also indicate that autophagy is essential for protecting cardiomyocytes from MI/R injury.

Although there are a large amount of studies that emphasize the crucial role of autophagy in cardiomyocytes under baseline conditions or stress, the precise mechanism of autophagy in cardiomyopathy remains elusive. The cytoskeleton and cytoskeleton-related proteins in cardiomyocytes are essential to ensure proper cardiac function, and cytoskeletal changes are an important cause of contractile dysfunction and cardiac remodelling [33,55]. So, we speculated whether the disruption of autophagy-induced cardiac hypertrophy and contractile dysfunction were related to cytoskeleton regulation. While most studies regarding the relationship between cytoskeleton and autophagy have focused on the functional role of the cytoskeleton in autophagosome formation and transportation [34,35], few studies have investigated how autophagy regulates cytoskeletal organization. The ciliopathy protein OFD1 (oral-facial-digital syndrome 1) and mechanically damaged cytoskeleton proteins were found to be degraded through autophagy [15,56]. Recently, we found autophagy could regulate cytoskeleton organization via degradation of a negative regulator of cytoskeleton organization, CLP36, to facilitate the renovation of spermatids and ectoplasmic specialization assembly in Sertoli cells [36]. CLP36 is a member of the PDZ and LIM protein family, which contains an N-terminal PDZ domain and a C-terminal LIM domain [57]. It is expressed throughout the developing heart and interacts with FHL1 (four and a half LIM domains protein 1) in adult cardiomyocytes, which has been implicated in muscle development, structural maintenance and signalling [58–60]. CLP36 could also function as a cytoskeletal organization scaffold and an adaptor for the recruitment of α-actinin-1 and palladin to form stress fibres [37]. Ridley & Hall [61] showed that once Swiss 3T3 fibroblasts were starved by serum deprivation for a few hours, the pre-existing stress fibres and focal adhesions disappeared, suggesting that they might be degraded by the autophagy–lysosome pathway. In this study, we find that CLP36 is also accumulated in *Atg7*-deficient cardiomyocytes, which indicates that autophagy may participate in maintaining cardiomyocytes' contractile function by regulating CLP36.

Stress fibres are commonly observed in many CVDs [23,24], and many mutations in stress fibre component proteins have been identified to be related to CVDs [25,26]. Many stress fibre component proteins are localized on Z-disc, which reveals cross-linked filament arrays that transmit tension and house myriads of proteins with diverse functions. Mutations in the genes for these proteins often lead to muscle diseases and cardiomyopathies [62]. The CLP36 accumulated and localized on the Z-disc, which might be recruited by or recruit other stress fibre component protein, such as palladin or α-actinin (figure 3*d–f* and 4*e–g*; electronic supplementary material, figures S1 and S2). The accumulation of the stress fibre on the Z-disc may induce sarcomere disorganization and influence the contractility of cardiomyocytes (figures 5 and 6*c*).

Cardiac fibrosis is characterized by net accumulation of extracellular matrix in the myocardium, and contributes to both systolic and diastolic dysfunction in many cardiac pathophysiologic conditions [63]. Cardiomyocyte death or injurious stimuli (such as pressure overload or myocardial inflammation) is the initial event responsible for activation of fibrogenic signals in the myocardium. In all conditions associated with cardiac fibrosis, fibroblast transdifferentiation into secretory and contractile myofibroblasts is the key cellular event that drives the fibrotic response [39]. At the earliest stages of reparative or fibrotic responses, myofibroblasts exhibit stress fibres composed of cytoplasmic actins [64]. The accumulation of CLP36 and some other stress fibre components in *Atg7*-deficient mice after ischaemia-reperfusion treatment may also be related to severe cardiac fibrosis. On the other hand, severe cardiac fibrosis in *Atg7*-deficient mice also indicates that autophagy is necessary for protecting against cardiomyocyte death during MI/R. These possibilities still require further studies in the future.

## 4. Material and methods

### 4.1. Animals

The *Atg7^flox/flox^* mouse strain (RBRC02759) [29] was purchased from the RIKEN BioResource Center with permission from Dr Masaaki Komatsu. The *Atg7^flox/flox^ MERCreMER* mice were bred from *Atg7^flox/flox^* mice and *MERCreMER* mice [30]. We administered an intraperitoneal injection of 0.225 mg g^−1^ (body weight) of tamoxifen (Sigma, T5648) or vehicle to eight-week-old mice once per day for 3 days. The surgical procedures were performed as described in previous studies [65]. Briefly, after anaesthetizing 8–10-week-old mice with chloral hydrate (400 mg kg^−1^ body weight, i.p.), we cannulated the trachea of mice with a polyethylene tube connected to a respirator with a tidal volume of 0.6 ml (110 breaths min^−1^). The heart was manually exposed through a small incision, and a slipknot was made around the left anterior descending coronary artery at 2–3 mm from its origin using a 7–0 silk suture. After 60 min of ischaemia, the slipknot was released, and then myocardium was reperfused. Mice that fully recovered from the surgical procedure were returned to standard animal housing conditions.

### 4.2. Antibodies

The mouse anti-ATG7 monoclonal antibody (SAB4200304), mouse anti-α-actinin monoclonal antibody (A5044) and rabbit anti-LC3B polyclonal antibody (L7543) were purchased from Sigma-Aldrich (St Louis, MO, USA). The rabbit anti-LC3 polyclonal antibody (ab128025) and rat anti-LAMP2 monoclonal antibody (ab13524) for immunofluorescence were purchased from Abcam (Cambridge, MA, USA). The rabbit anti-SQSTM1/p62 polyclonal antibody (5114) was purchased from Cell Signaling Technology (Danvers, MA, USA). The rabbit anti-CLP36 antibody (11674-1-AP) was purchased from Proteintech Group (Chicago, IL, USA). The mouse anti-palladin monoclonal antibody (NBP1-25959) was purchased from Novus Biologicals (Littleton, CO, USA). The GAPDH (ab1019t) antibody was purchased from Bo Ao Rui Jing (Beijing, China). The goat anti-rabbit TRITC, goat anti-rabbit FITC and goat anti-mouse FITC-conjugated secondary antibodies were purchased from Zhong Shan Jin Qiao (Beijing, China). The FITC-phalloidin (40735ES75) was purchased from YEASEN (Shanghai, China). The Alexa Fluor 680-conjugated goat anti-mouse and the Alexa Fluor 680-conjugated goat anti-rabbit secondary antibodies for immunoblotting were purchased from Invitrogen (Carlsbad, CA, USA).

### 4.3. Immunoblotting

Heart extracts were prepared in cold RIPA-like buffer (25 mM Tris HCl, pH 7.6, 150 mM NaCl, 2 mM EDTA, 1% NP-40, 1% sodium deoxycholate, 0.1% sodium dodecyl sulfate, 1 mM phenylmethylsulfonyl fluoride and a protein inhibitor cocktail; 04693132001, Roche Diagnostics, Basel, Switzerland) for 30 min on ice after sonication. The homogenates were centrifuged at 12 000 r.p.m. for 15 min at 4°, and the protein concentrations were determined by the Bio-Rad protein assay. The protein lysates (approx. 25 µg) were electrophoresced under reducing conditions in SDS-PAGE gels and transferred onto nitrocellulose membranes. After incubating in primary antibody, immunoblotting was performed using a fluorescent dye-labelled secondary antibody (Invitrogen), and the blots were scanned using an Odyssey infrared imager.

### 4.4. Immunofluorescence

Hearts were dissected from mutant and control mice immediately after euthanasia, fixed in 4% PFA (P1110, Solarbio, Beijing, China) at room temperature for up to 24 h, stored in 70% (vol/vol) ethanol, and embedded in paraffin. The 5 μm sections were prepared and mounted on glass slides. After deparaffinization, sections were boiled for 15 min in sodium citrate buffer for antigen retrieval. For phalloidin and CLP36, hearts were fixed in 4% PFA at room temperature for 4 h and dehydrated in 30% sucrose. Then, the tissue was embedded in optimum cutting temperature compound (OCT, 4583, Tissue-Tek, Torrance, CA, USA) and cut into 6 µm sections using a microtome-cryostat (Leica CM1950). After washing with PBS three times and blocking with 5% bovine serum albumin (BSA), the primary antibody was added to the sections and incubated at 4°C overnight, followed by incubation with the secondary antibody. The nuclei were stained with 4′,6-diamidino-2-phenylindole (DAPI). The images were taken immediately using a TCS SP8 microscope (Leica) and an Eclipse Ti-S inverted microscope (Nikon).

### 4.5. Echocardiography

The echocardiography analysis in animals was performed as described before [17]. Images were obtained using Vevo 770 (Visualsonics, CA, USA) in M-mode with a 12 MHz microprobe. The mice were lightly anaesthetized using 1.5% isoflurane and restrained on a heated imaging table, and hairs on the chest were removed. Images were obtained from M-mode of the parasternal short-axis view. All values were averaged over five consecutive cardiac cycles and measurements were analysed by two independent researchers blinded to the treatment status.

### 4.6. Tissue collection and histological analysis

Hearts from at least three mice for each genotype were dissected immediately after euthanasia, fixed in 4% (mass/vol) paraformaldehyde (PFA) for up to 24 h, stored in 70% (vol/vol) ethanol and embedded in paraffin. The 5 µm sections were prepared and mounted on glass slides. After deparaffinization, slides were stained with H&E for histological analysis and Sirius red for cardiac fibrosis analysis.

### 4.7. Real-time quantitative PCR (qPCR) analyses

Total RNAs were isolated from mice hearts as previously described [66]. cDNA was synthesized by using the PrimeScriptTM RT Reagent Kit (TaKaRa, RR037A). cDNA (10 ng) mix was added to KAPA SYBR FAST Master Mix (KAPA Biosystems, USA) with specific primer sets (*Clp36* forward: 5′-CCACATCCTTCCTGGTTCTG3′; and reverse: 5′-TGGTGATCCCTCAGCTT CAC-3′; *Gapdh* forward: 5′- GGTGGTGCTAAGCGTGTTAT-3′; and reverse 5′-ACCTCTGT CATCTCTCCACA-3′). The PCR was carried out with the Roche Light Cycler 480II System and the results were analysed using the LightCycle480SW 1.5.1.

### 4.8. Transmission electron microscopy

The mouse hearts were dissected and fixed with 2.5% glutaraldehyde and 2% PFA in 0.2 M cacodylate buffer overnight. The tissues were immersed in 1% OsO_4_ in 0.2 M cacodylate buffer for 1 h at 4°C. Then, the samples were dehydrated through a graded ethanol series and embedded in resin. Ultrathin sections were cut on an ultramicrotome, stained with uranyl acetate and lead citrate, and observed using a JEM-1400 TEM.

### 4.9. Statistical analysis

All data are presented as the mean ± s.e.m. The statistical significance of the differences between the mean values for the different genotypes was measured by Student's *t-*test with a paired, two-tailed distribution. The data were considered significant when the *p-*value was less than 0.05 (*).

## Supplementary Material

Figure S1. Immunofluorescence analysis of CLP36. Figure S2. CLP36 co-localizes with a-actinin.
