# Supplementary material for: Autophagy protects cardiomyocytes from the myocardial ischaemia-reperfusion injury through the clearance of CLP36
Source: Open Biol. 2016 Aug 10;6(8):160177. doi: 10.1098/rsob.160177 (PMC5008017; doi:10.1098/rsob.160177)
Supplement: Figure S1. Immunofluorescence analysis of CLP36. Figure S2. CLP36 co-localizes with a-actinin. [file rsob160177supp1.doc]

**Supplementary material**

**Autophagy protects cardiomyocytes from the** **myocardial ischemia-reperfusion injury through the clearance of CLP36**

Shiguo Li^1^ ^#^*,Chao Liu^2,4#^, Lei Gu^3,4#^, Lina Wang^2,4^, Yongliang Shang^2,4^, Qiong Liu^1^, Junyi Wan^1^, Jian Shi^2,4^, Fang Wang^2,4^, Zhiliang Xu^2,4^, Guangju Ji^3^*, Wei Li^2,4^*

^1^ Department of Radiology, State Key Laboratory of Cardiovascular Disease, Fuwai Hospital, National Center for Cardiovascular Diseases, Chinese Academy of Medical Sciences and Peking Union Medical College, Beijing 100037, R.P. China

^2^ State Key Laboratory of Stem Cell and Reproductive Biology, Institute of Zoology, Chinese Academy of Sciences, Beijing 100101, R.P. China

^3^ National Laboratory of Biomacromolecules, Institute of Biophysics, Chinese Academy of Sciences, Beijing 100101, R.P. China

^4^ University of Chinese Academy of Sciences, Beijing 100049, R.P. China

# These authors contributed equally to this work.

# * Correspondence should be sent to:

# Dr. Wei Li

Institute of Zoology, Chinese Academy of Sciences

1 Beichen West Road, Chaoyang District,

Beijing 100101, P.R. China

Email: **leways@ioz.ac.cn**

Tel: 86-10-64807529

# Dr. Guangju Ji

Institute of Biophysics, Chinese Academy of Sciences

15 Datun Road, Chaoyang District,

Beijing 100101, P.R. China

Email: **gj28@ibp.ac.cn**

Tel: 86-10- 64846720

# Dr. Shiguo Li

Fuwai Hospital, Chinese Academy of Medical Sciences

North Lishi Road, Xicheng District,

Beijing 100037, P.R. China

Email: [**sgli2000@hotmail.com**](mailto:sgli2000@hotmail.com)

**Key words**: autophagy, *Atg7*, myocardial ischemia-reperfusion injury, CLP36, stress fiber.

**Running title**: Autophagy protects against ischemia-reperfusion injury.

**Supplementary Figures**

**
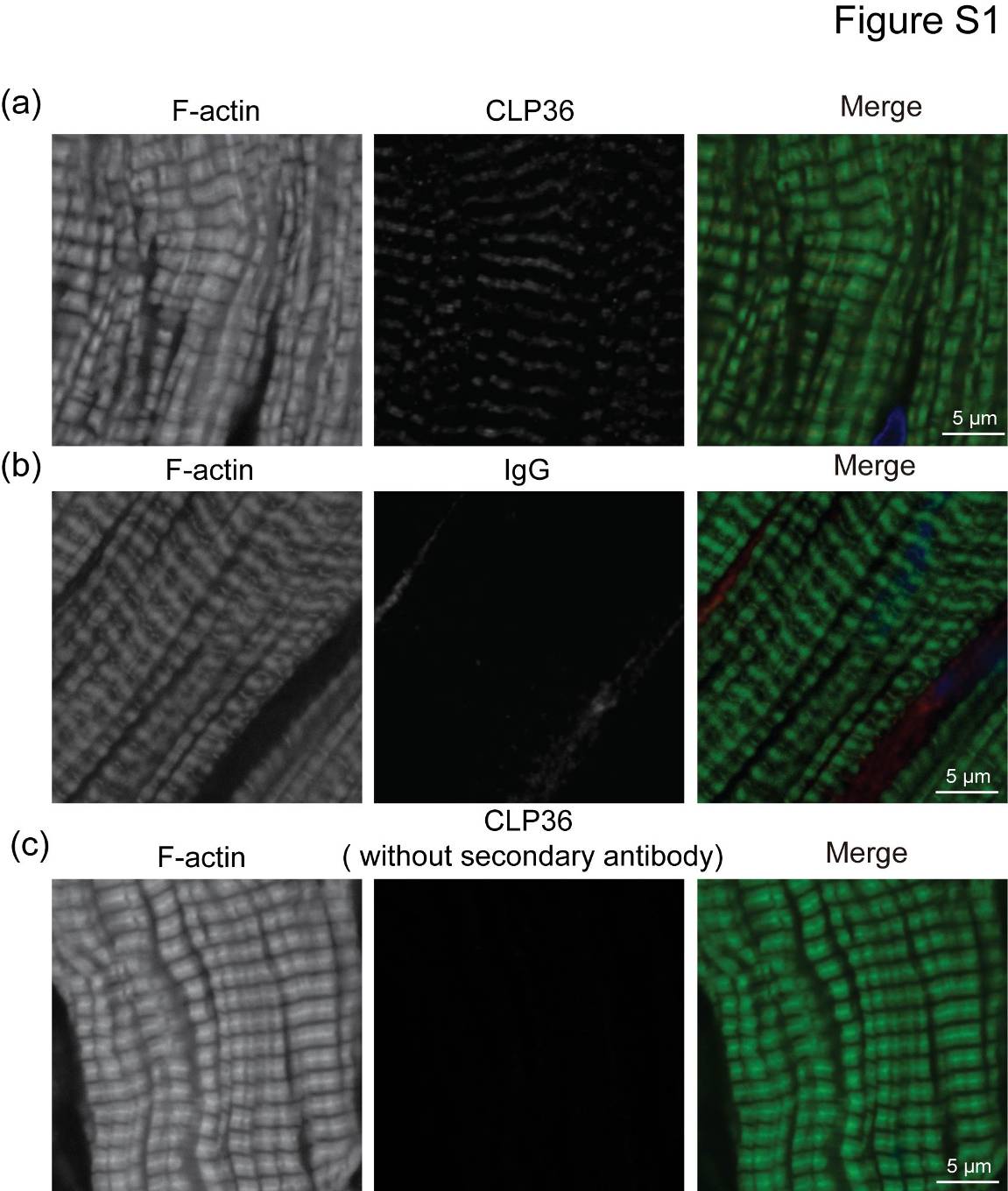
**

**Figure S1.** **Immunofluorescence analysis of CLP36.**

(a) Immunofluorescence analysis using phalloidin (green, labeled by FITC) and CLP36 (red) were performed in mouse cardiomyocytes. Nuclei were stained with DAPI (blue).

(b) Immunofluorescence analysis using phalloidin (green, labeled by FITC) and rabbit IgG (red) were performed in mouse cardiomyocytes. Nuclei were stained with DAPI (blue).

(c) Immunofluorescence analysis using phalloidin (green, labeled by FITC) and CLP36 (without secondary antibody) were performed in mouse cardiomyocytes. Nuclei were stained with DAPI (blue).

**
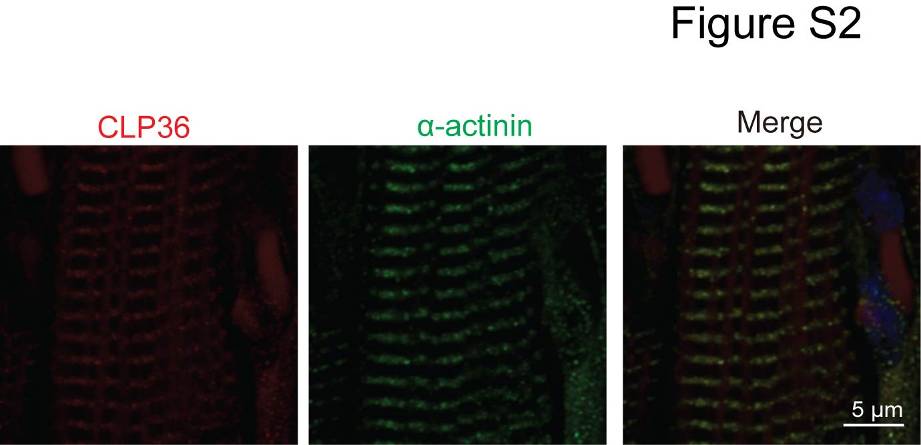
**

**Figure S2.** **CLP36 co-localizes with α-actinin.**

Immunofluorescence analysis usingα-actinin (green) and CLP36 (red) were performed in mouse cardiomyocytes. Nuclei were stained with DAPI (blue).
